# Impact of RNA-seq attributes on false positive rates in differential expression analysis of de novo assembled transcriptomes

**DOI:** 10.1186/1756-0500-6-503

**Published:** 2013-12-03

**Authors:** Emmanuel González, Simon Joly

**Affiliations:** 1 Institut de recherche en biologie végétale, Université de Montréal, 4101 Sherbrooke E, Montréal, H1X 2B2, (QC), Canada; 2 Montreal Botanical Garden, 4101 Sherbrooke E, Montréal, QC H1X 2B2, Canada

**Keywords:** *de novo* transcriptome assembly, Differential gene expression, Differential isoform expression, Non-model organisms, False positive rates, RNA-seq

## Abstract

**Background:**

High-throughput RNA sequencing studies are becoming increasingly popular and differential expression studies represent an important downstream analysis that often follow de novo transcriptome assembly. If a lot of attention has been given to bioinformatics tools for differential gene expression, little has yet been given to the impact of the sequence data itself used in pipelines.

**Results:**

We tested how using different types of reads from the ones used to assemble a de novo transcriptome (both differing in length and pairing attributes) could potentially affect differential expression (DE) results. To investigate this, we created artificial datasets out of long paired-end RNA-seq datasets initially used to build the assembly. All datasets were compared via DE analyses and because all samples come from the same sequencing run, DE of genes or isoforms can be interpreted as false positives resulting from sequence attributes. If the false positive rate for differential gene expression does not seem to be strongly affected by sequencing strategy (max. of 3.5%), it could reach 12.2% or 28.1% for differential isoform expression depending of the pipeline used. The effect of paired-end vs. single-end strategy was found to have a much greater impact in terms of false positives than sequence length.

**Conclusion:**

In light of false positive rate results, we recommend using paired-end over single-end sequences in differential expression studies, even if the impact is less serious for differential gene expression.

## Background

Recent advances in massively parallel sequencing technologies have created huge opportunities in the field of transcriptomics [1-4]. High-throughput RNA sequencing, or RNA-seq, together with the development of powerful computational methods, have given researchers the opportunity to study non-model organisms by assembling *de novo* transcriptomes, i.e. without a reference genome or transcriptome [5-7]. Such computational methods are being increasingly used as species for which a referenced genome or transcriptome exists represent a tiny fraction of all species.

Beyond transcriptome assembly, one of the great advantages of RNA-seq is to allow gene or isoform expression quantification and the evaluation of their differential expression under different conditions. By providing direct quantification of all transcripts of a sample, RNA-seq data has the potential to overcome several limitations of previous approaches that were limited to small scaled studies (quantitative PCR) or that required important genomic knowledge and resources (microarrays) [8]. For instance, RNA-seq data has been rapidly found to outperform microarrays approaches [9].

Despite these great promises, accurate and reliable differential expression studies are still challenging and a lot of attention has been given to bioinformatics tools [10,11]. These challenges involve, for example, mapping sequencing reads to a reference transcriptome and statistically testing for differential expression. Yet, surprisingly little attention has been given to the impact of sequence types used as input on differential expression analyses. Practically, differential expression studies requires two decisions in terms of sequencing parameters, i.e. RNA-seq reads length and the possibility of sequencing RNA fragments from one end (single-end reads) or both ends (paired-end reads). Of course, long paired-end reads are the best choice for constructing an assembly as they outperform single-end alignment in terms of both sensitivity and specificity [12]. However, single-end reads might be favored for economic reasons, especially for gene quantification as an increase in sensitivity and specificity might not always be required. For instance, although paired-end reads are generally thought to be imperative for correctly estimating isoform-specific expression, they might not be required for gene expression. As such, it could seem appropriate to assemble a transcriptome using paired-end reads, but to use single-end reads when quantifying gene expression on biological replicates. This common belief has rarely been tested and little is known about the impact of sequencing decisions on differential expression results accuracy.

The objective of this study is to test how sequencing strategies (i.e. sequencing length and pairing options) affects false positive rates in differential expression results at both gene and isoform level. We created different types of sequence datasets from a long paired-end sequencing run and analyzed the datasets with each other to evaluate the impact of the sequence type on false positive rates. Our results show that the choice of sequence used in differential gene expression studies can sometimes have an important impact on the accuracy of the results.

## Results and discussion

### Transcriptome assembly

Two samples consisting of different tissues from distant plants were studied: RNA was extracted from willow (*Salix purpurea* L.; Salicaceae; Rosids) buds, as well as from all stages of flower development of *Rhytidophyllum vernicosum* Urb. & Ekman (*Gesneriaceae*; Asterids) using a modified CTAB method [13]. Both samples were sequenced using 100 bp paired-end strategy on an Illumina Hiseq 2000 sequencer at the Genome Quebec Innovation Centre (Montreal, Canada). After removing poor quality sequences and nucleotides, a *de novo* transcriptome was built with Trinity [4]. To evaluate whether different types of sequences used in the transcriptome assembly could affect false positive rates in DE analyses, we assembled two transcriptomes, that is with and without pairing information (Table 1).

**Table 1:** Raw data and trinity assemblies statistics

	**Paired-end assembly**		**Single-end assembly**	
	***Salix purpurea***	***Rhytidophyllum vernicosum***	***Salix purpurea***	***Rhytidophyllum vernicosum***
**Number of sequences**	72,121,862	74,653,202	72,121,862	74,653,202
**Isoforms**	185,052	165,516	157,076	135,863
**Genes**	92,450	70,662	86,087	66,261
**Transcriptome length (bp)**	249,828,609	289,064,658	192,652,014	214,836,625
**N50**	2,235	2,800	2,091	2,583

### Bioinformatics pipelines

**Figure 1:**
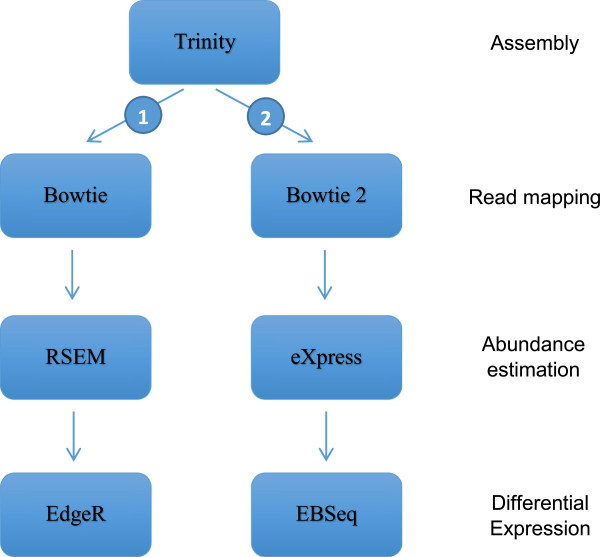
The two different bioinformatic pipelines used to assemble RNA-seq reads and analyze gene and isoform differential expressions.

Starting from the transcriptome assemblies, two different pipelines were used to calculate contig abundances and differential expressions (Figure 1). The first one is directly available from within the Trinity suite and is composed of the ungapped aligner Bowtie [14], RSEM [15] for calculating transcript abundances, and EdgeR [16] for testing differential expression. Trinity being widely used, we thought it was relevant to test this pipeline that might represent the default option in many studies. The second pipeline used Bowtie2 [17], eXpress [18] for estimating transcripts abundances, and EBSeq [19] for differential expression. This pipeline is based on a gapped aligner known to perform well [20] and recommended for use with eXpress [18]. One advantage of this second pipeline is speed: bowtie2 + eXpress is very fast compared to Bowtie + RSEM as implemented in Trinity pipeline. We decided not to use EdgeR in the second pipeline as EdgeR requires setting a dispersion factor that was hard to evaluate for our artificial samples. EBSeq is an empirical Bayesian approach that directly models differential variability as a function of the number of isoforms, providing a good approach for isoform level inference. No assumption has to be made as the expectation–maximization algorithm is used for estimating all parameters through an iterative procedure until convergence. EBSeq is also capable to identifying DE genes. We acknowledge that several other pipelines could have been tested. Yet, evaluating pipelines is beyond the scope of this study that focuses on the impact of sequence type. We chose two different pipelines to make sure they did not affect our results.

### Short-read sequences mapping

To simulate 50 base pairs paired-end (50 bp PE), 100 bp single-end (100 bp SE) and 50 bp single-end (50 bp SE) reads, initial 100 bp PE RNA-seq reads were trimmed or pairing information removed. Each different set of reads was mapped back to the *de novo* transcriptomes (Table 2). The ca. 10% difference between paired-end and single-end mapping can be explained by the fact that single-end mapping doesn’t have any pairing constraint. Paired-end back mapping percentages are thus lower as any repetitive short read sequence should be placed more reliably since its mate contributes to mapping information. Both species showed similar back mapping percentages between assemblies built from paired-end reads and assemblies built from single-end reads (PE and SE assemblies; Table 2), although statistics show that the PE assembly is substantially larger than the SE assembly (Table 1). Furthermore, genes and isoforms are longer in PE assembly compared to SE assembly. This supports previous observations that PE assembly outperforms SE assembly in terms of sensitivity and specificity [12].

**Table 2:** **Percentage of initial reads mapped back to the *de novo *** **transcriptome**

		**100PE**	**50PE**	**100SE**	**50SE**
***Salix purpurea***	**PE assembly**	83%	81%	90%	89%
	**SE assembly**	83%	81%	91%	90%
***Rhytidophyllum vernicosum***	**PE assembly**	88%	88%	95%	95%
	**SE assembly**	89%	88%	95%	95%

PE assembly and SE assembly relate to the transcriptomes assembled with paired-end or single-end reads respectively.

### Differential expression analyses

**Figure 2:**
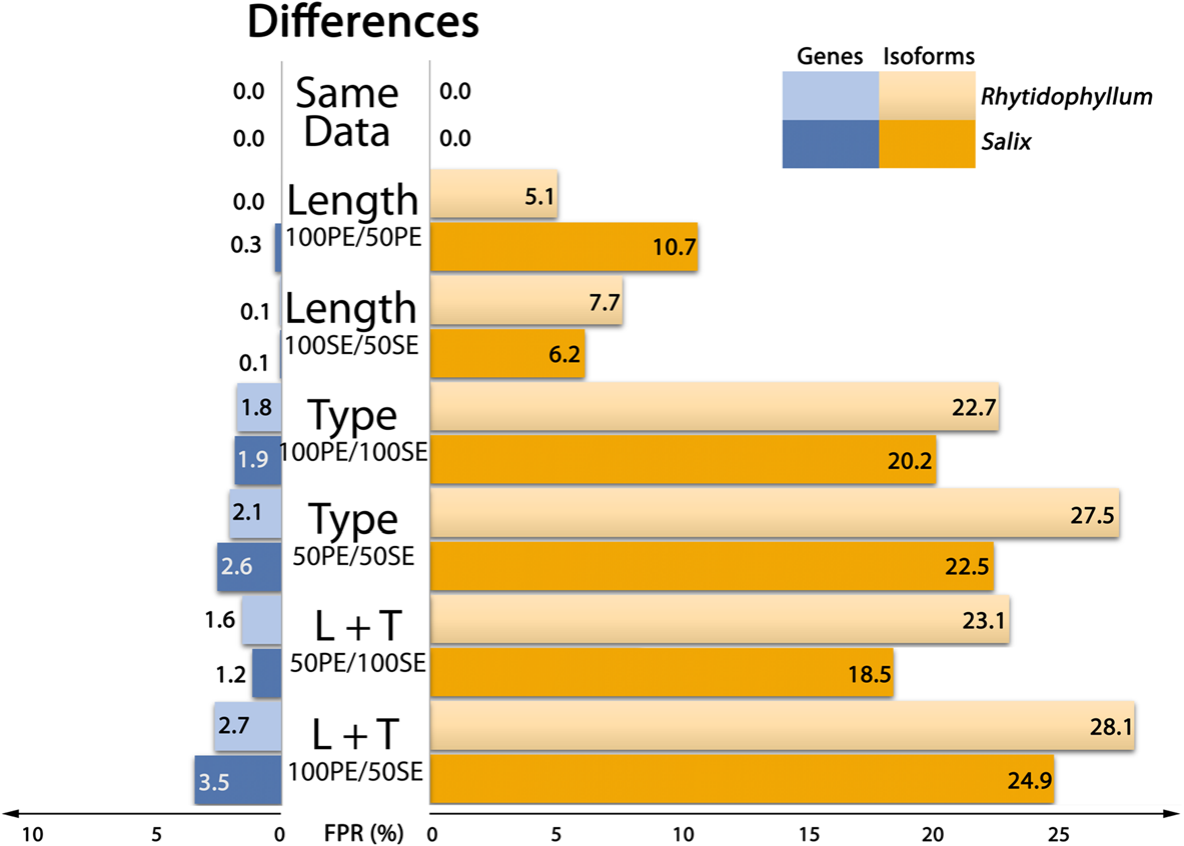
False positive ratios in DE experiment (pipeline 1) as a function of input data type.

**Figure 3:**
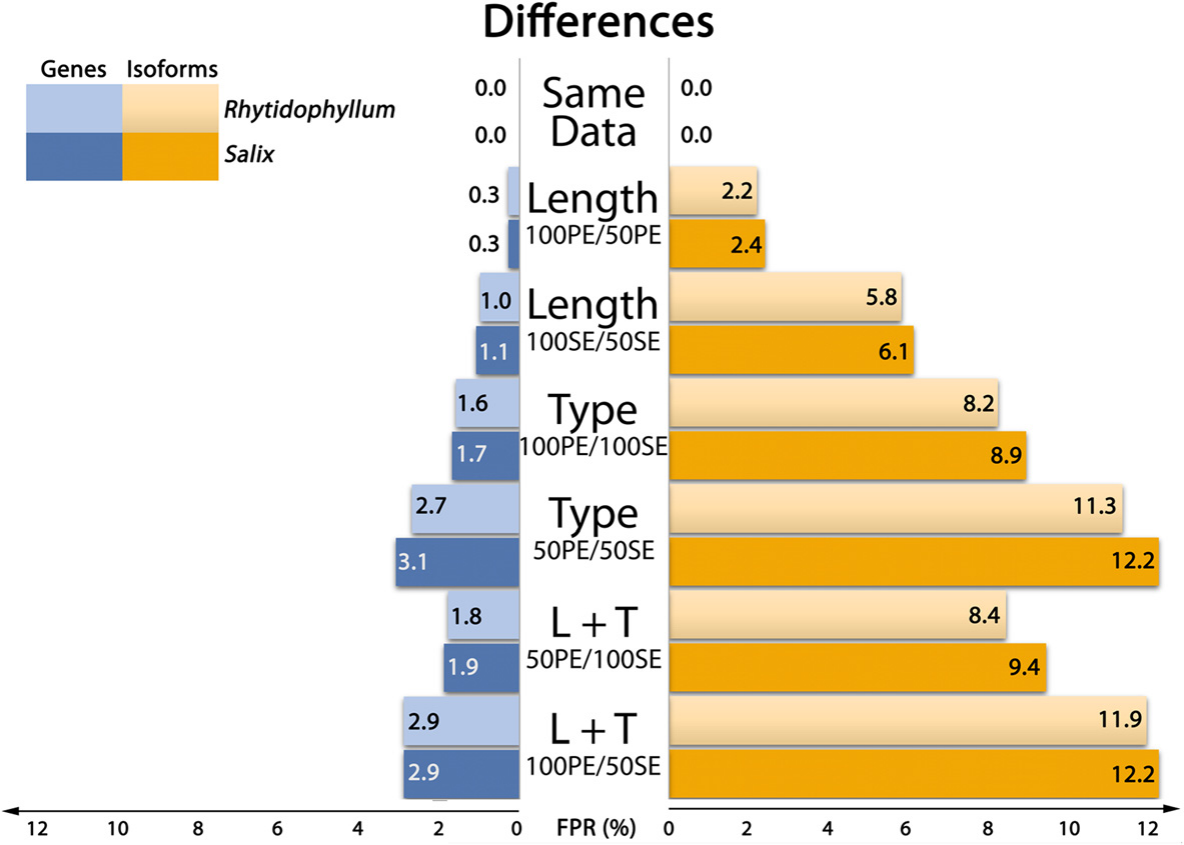
False positive ratios in DE experiment (pipeline 2) as a function of input data type.

Because we are testing differential gene or isoform expressions of a sample with itself, the expectation is to find no differential gene expression. Indeed, if any read is distinctive enough from others, it should be unambiguously mapped back to the transcriptome. If pairing type or sequence length would not affect the reads specificity, the different datasets should have exactly the same genes and isoforms abundance and no differential expression should be observed. As shown in Figures 2 and 3, only sets that are exactly of the same length and pairing type show this trend (“same data” line). False positives appear when modified RNA-seq datasets are compared. Any observation of differential gene expression means that strategies between data sets affect the abundance estimates.

Comparing Figures 2 and 3 shows that pipeline strategies do not have much impact on DE at gene level: differences observed in false positive rates are minor. Interestingly, these results suggest that the use of a gapped alignment, which may seem unnecessary as reads are directly mapped to transcripts, does not affect the results. In fact, Bowtie2 showed extremely good results when coupled to eXpress, the latter handling multimapping very well. At the isoform level, on the contrary, the second pipeline clearly outperforms the first one (Figures 2 and 3). This result is not totally unexpected as EBSeq approach was developed mainly for isoform differential expression studies [19]. Because pipeline 2 gives better results at the isoform level compared to pipeline 1, only results from this pipeline will be discussed in the following for concision and clarity purposes. However, the pipeline choice does not affect our conclusions.

### Sequence length

In both species, reducing sequence length had little impact on gene expressions (Figure 3, “Length” lines): FPR adds up to 0.3% for PE sequences and about 1% for SE sequences. In the isoform approach, FPR gets approximately six times higher: about 2% for PE sequences and 6% for SE sequences. Sequence length influences FPR as shorter reads have potentially more multimapping events than longer reads as they are less specific. This uncertainty results in different estimated counts for genes or isoforms compared to longer sequences.

### Paired-end vs. single end reads

To investigate the effect of reads pairing in differential expression analyses, DE was tested between samples in which counts were estimated with paired-end sequences (100 and 50 bp) to samples in which counts were estimated with single-end sequences (100 and 50 bp). Gene DE analysis of paired versus unpaired sequences resulted in FPR of 1.7% and 1.6% (100 bp) and 3.1% and 2.7% (50 bp), for *Salix* and *Rhytidophyllum* respectively (Figure 3, “Type” lines). Whereas shortening sequence lengths had little consequences in gene DE analyses, removing pairing attributes had more impact on FDR. As for sequence length, isoform DE is more affected in terms of FDR: for *Salix,* FPR reaches 8.9% (100 bp) and 12.2% (50 bp), while it is 8.2% (100 bp) and 11.3% (50 bp) for *Rhytidophyllum*. Again, shorter hence less specific sequences result in higher FPR.

Lastly, DE results between datasets that varied in both sequence types and lengths produced the expected results given the previous observations on length and pairing attributes. The highest FPR were observed when longest PE sequences (100 bp) were tested against the shortest SE sequences (50 bp). Both *Rhytidophyllum* and *Salix* showed a FPR of 2.9% for gene analysis and close to 12% for isoform analysis (Figure 3, “L + T” lines). When shorter PE sequences (50 bp) were tested against longer SE sequences (100 bp), false positive rates were slightly lower both for genes (1.8% and 1.9% for *Rhytidophyllum* and *Salix*, respectively) and isoforms (8.4% and 9.4% for *Rhytidophyllum* and *Salix*, respectively).

**Figure 4:**
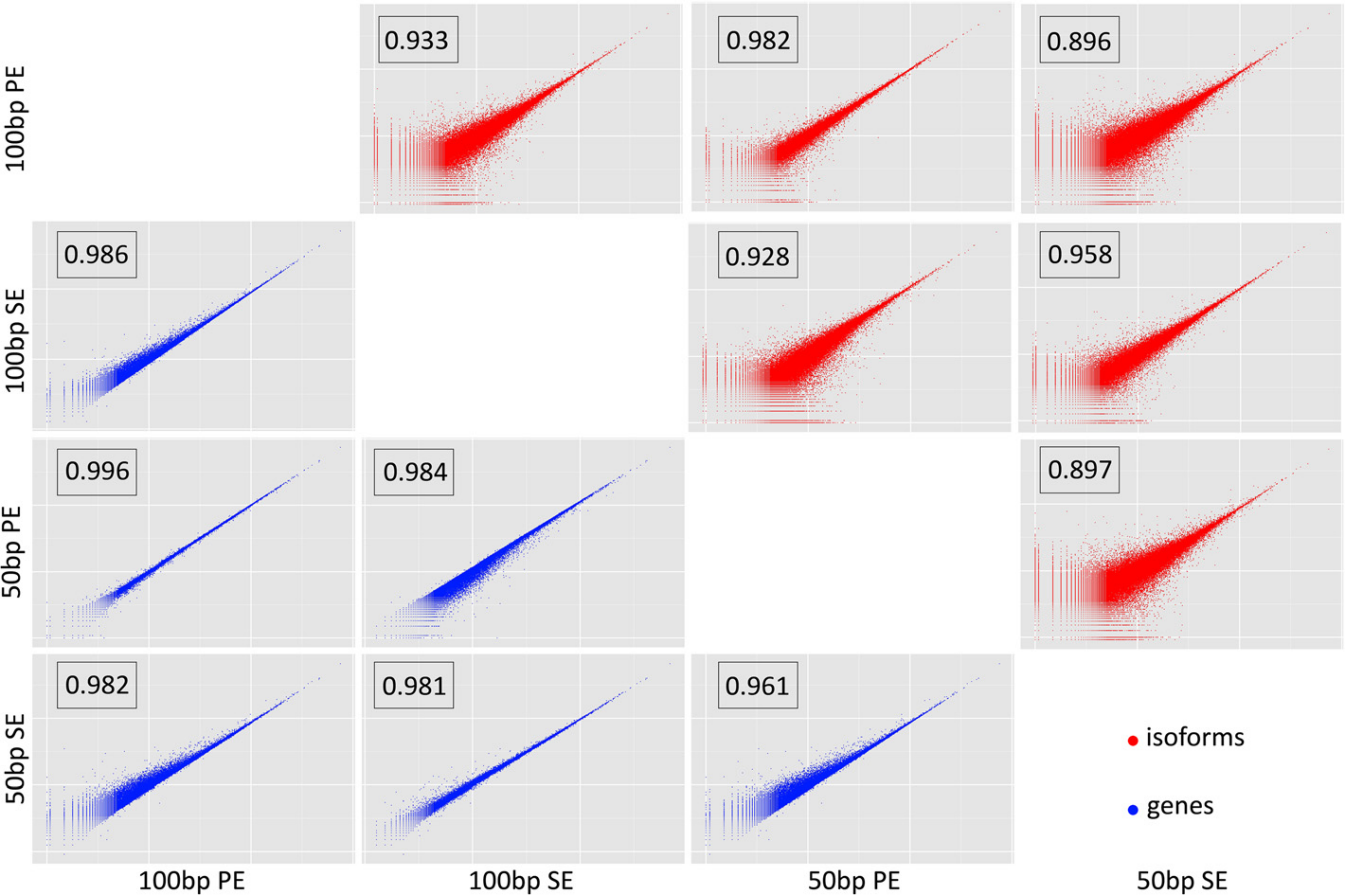
**Scattered plots of isoform (red) and gene (blue) log-transformed expression between all *Salix purpurea *sequence sets.** The numbers indicate the Pearson correlation.

Although the main results are given in terms of FDR, the overall pattern is the same when considering the correlation in transcripts or genes counts for pairs of datasets (Figure 4). That is, scatterplots are more scattered and correlations smaller for isoforms than for genes, and the strong effect of sequence type can also be observed (Figure 4).

### Gene or isoform expression

FDR suggest that the impact of the type of sequence used on DE is greater for isoforms than for genes (Figures 2, 3). Overall, isoform DE analysis on *Salix* led to 4.6 times more false positives than gene DE analysis. Interestingly, very similar proportions were found for *Rhytidophyllum* (4.7 ×). These results are not really surprising. Indeed, mapping uncertainty resulting from smaller and SE sequences is expected to be most important between isoforms of a single gene and less rarely among genes. Consequently, it is reassuring that gene DE is less affected by sequence type. Nevertheless, one could argue that 1% of false positive is not completely trivial considering the number of genes involved in such analyses.

### Effect of the transcriptome assembly strategy

**Figure 5:**
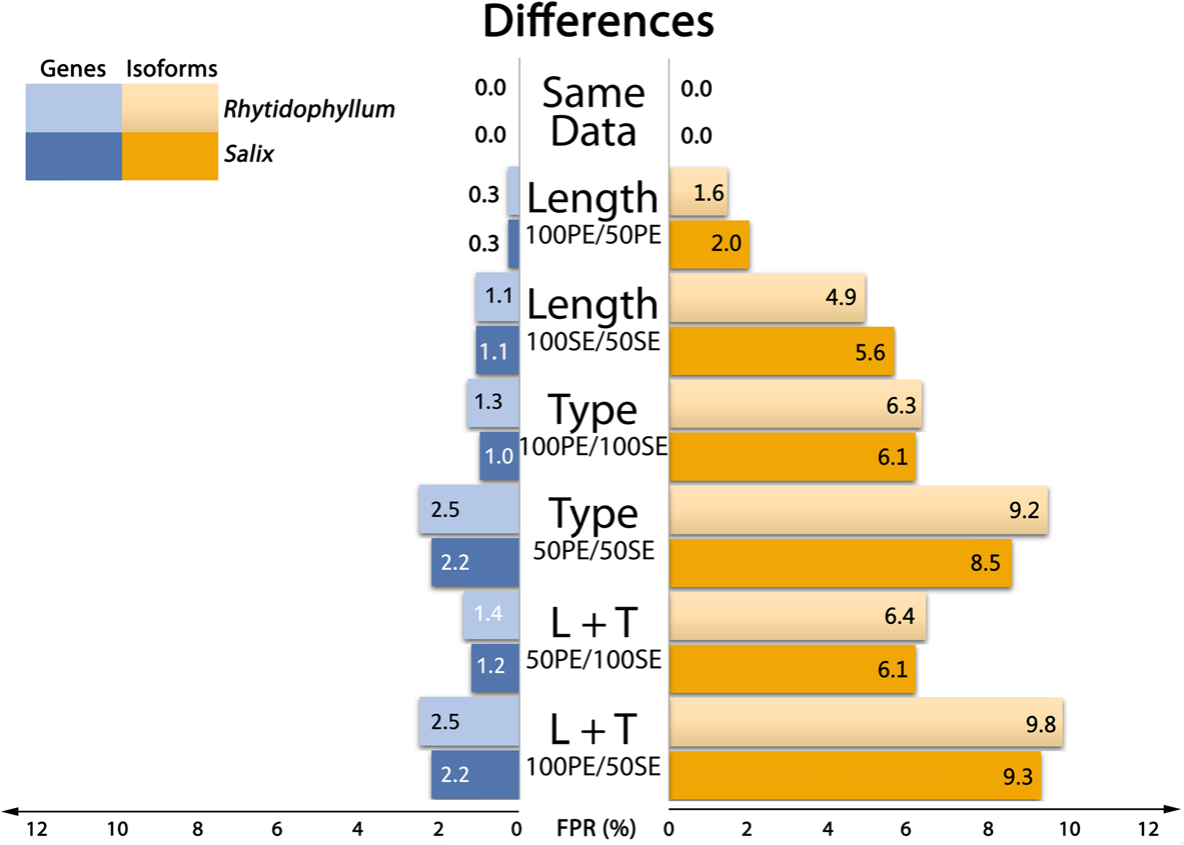
False positive rates in DE experiment (pipeline 2) as a function of input data type when abundance is estimated on the single-end assembly.

Our results show that FPR are at their highest when sequences of different pairing attributes are involved (Figures 2, 3; “Type” lines). Considering that our observations were so far based on a transcriptome that was assembled using PE sequences, an ensuing interrogation is whether the intrinsic PE nature of the assembly could inflate FDR for SE sequences datasets. We thus performed the same analyses as above on another *de novo* transcriptome assembled from 100 bp SE RNA-seq data using pipeline 2 (Table 1; Figure 5). The results obtained with this SE transcriptome are very similar to those described above (Figure 3): for both species, overall gene FPR are lower by a ratio of 0.008 and overall isoform FPR are lower by a ratio of 0.2. Moreover, both figures display a very similar profile, suggesting that the type of data used to assemble the transcriptome did not affect our results on FDR. The slight decrease of the FPR observed with the SE transcriptome assembly could potentially be attributed to its smaller size in terms of genes and isoforms relative to the PE assembly (Table 1), which is likely to decrease read mapping uncertainty.

### p-value threshold

**Figure 6:**
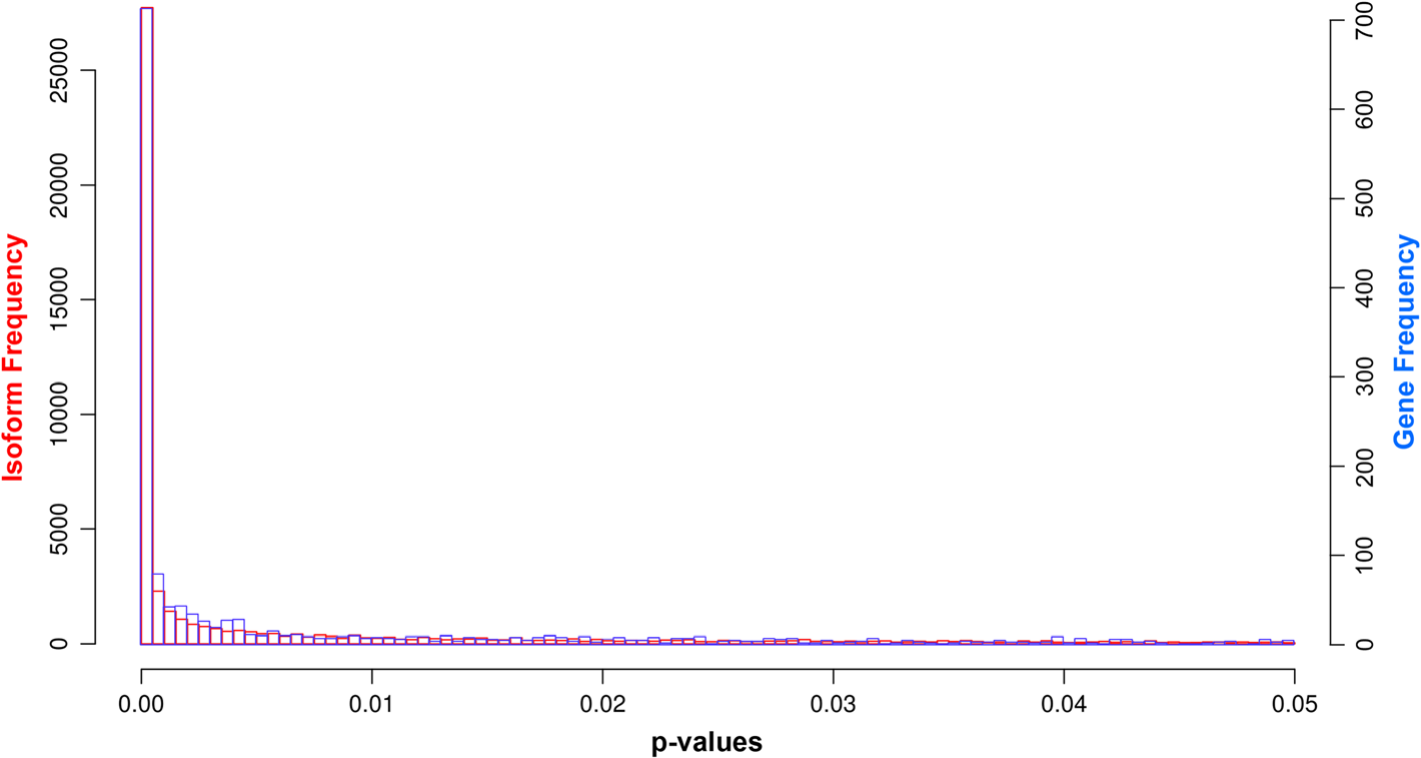
**Isoform (in red) and gene (in blue) p-values distribution.** Only values between 0 and 0.05 are represented.

Given that all previous calculations were based on a standard p-value of 0.05, we investigated whether this arbitrary threshold could affect our conclusions. This could be the case, for instance, if the significant results were always marginally significant; that is if p-value for significant genes mostly fell between 0.05 and 0.01. The distribution of p-values for all *Salix* isoforms and genes clearly show that a more conservative p-value would not affect our conclusions as the majority of the p-values were below 0.001 (Figure 6). The distribution of p-values for all *Rhytidophyllum* isoforms and genes, although not reproduced here, show the same trend.

### Limitations of the study

Our objective here was not to thoroughly explore all possible parameters that could affect DE analyses with respect to the sequencing strategy (e.g. sensitivity). Instead, we wanted to investigate whether DE analyses could be affected by the choice in sequencing strategy and broadly quantify this error. We thus acknowledge that there are limitations to the extrapolation of our conclusions to other conditions or organisms. A first limitation is related to the number of DE analyses pipelines investigated. Although more pipelines could have been explored, this was clearly not the aim of the study as aspects of different pipelines have been compared elsewhere [1,3,21-24]. Our approach was to use very distinct approaches to validate our results. Because the two pipelines gave similar results, we think the bioinformatics aspects of this study did not affect the main conclusions. Another limitation comes from the fact that only two plants have been studied and it consequently might be difficult to extrapolate our results to other organisms. Yet, because these two species diverged more than 100 mya [25] and because different tissues were used (buds and flowers), we think our results are probably quite general in plants and that they could even perhaps be extended to other eukaryotes that have similar transcriptome characteristics (e.g., size, isoform numbers, etc.). Finally, we purposely did not include any biological replicate. Such replicates are mandatory when analyzing differential expression as it allows distinguishing treatment effects from individual variance within treatments. Adding biological replicates would probably have resulted in finding fewer DE transcripts and genes in our analyses. But we deliberately chose to ignore any replicate because we want to solely observe the pure consequence of a given sequencing strategy. Hence, our results show the intrinsic error due to the sequence type in a DE experiment.

## Conclusions

With the limitations mentioned in the previous section in mind, our results show that the choice of sequence type does have an impact on differential expression results. Interestingly, we found that using single-end instead of paired-end sequences had a greater impact than reducing the length of the sequences from 100 bp to 50 bp (Figure 3). These results make sense because the paired-end information reduces uncertainty during read mapping. For instance, if a pair of reads is essential for the accurate mapping of this pair to one specific isoform of a gene, then disrupting the pair will result in one read being mapped ambiguously to two or more isoforms of the gene. This uncertainty results in biased estimated abundance frequencies of isoforms. For instance, Table 3 reports the abundance estimates for the four isoforms of one randomly chosen *Rhytidophyllum vernicosum* gene (i.e. Trinity cluster), which illustrates how relative abundances can differ between SE and PE datasets. The total number of transcript counts, which was found to be higher for SE than for PE datasets (Table 3), also highlights the greater uncertainty resulting from SE reads. The fact that such uncertainty in mapping occurs less frequently among genes likely explains why the FDR was less important for genes than for isoforms. Finally, the smaller impact of sequence length on FDR than pairing type can probably be explained because the distance covered on the transcript by the length reduction is less important than when pairing information is removed.

**Table 3:** ***Rhytidophyllum vernicosum *** **transcript counts for a gene and total number of transcript counts for 100 bp PE and 50 bp SE sequence datasets**

	**100 bp PE counts**	**50 bp SE counts**
**Isoform 1**	0 (0%)	79 (21%)
**Isoform 2**	3 (1%)	1 (0%)
**Isoform 3**	47 (17%)	84 (22%)
**Isoform 4**	219 (81%)	210 (56%)

Numbers represent raw counts and relative frequencies are given in parentheses.

Overall, our results suggest that paired-end sequence is relatively crucial for obtaining precise isoform differential expression. We also suggest using paired-end sequences for gene DE, even though this is less critical. Indeed, the mapping uncertainty remains important for gene count estimation with single-end sequences: comparisons of counts obtained with paired-end vs. single-end resulted in almost 2% false positives. If someone is to make economies, the best solution seems to be to sequence 50 bp paired-ends rather than 100 bp paired-ends (for fragments of the same length). This approach seems to result in very small differences in count estimation for both genes and isoforms.

## Methods

### Transcriptome assembly

Prior to assembling reads, Trimmomatic [26] was used to remove bad quality Illumina RNA-seq data and trim poor quality nucleotides at the beginning and the end of each sequence. The following parameters were used in the command line: LEADING:15 TRAILING:15 SLIDINGWINDOW:5:15 MINLEN:40. For the assembly, Trinity software [4] was used to reconstruct the transcriptome *de novo* using default settings. Gene sequences were obtained using isoform union method consisting on qualifying as “gene”, the union of transcripts identified by Trinity as isoforms of the same gene.

### Read mapping

We used two different approaches to map RNA-seq reads to the reference transcriptome: Bowtie [14] that maps reads to a reference without allowing any gap, and Bowtie2 [17] that allows gaps during mapping. Bowtie was run as a part of Trinity pipeline. The following parameters were used in the command line: alignReads.pl --left R1.fastq --right R2.fastq --target Trinity.fasta --seqType fq --aligner bowtie --max_dist_between_pairs 800 -- -p 16. In Bowtie 2, the following parameters were used: Bowtie2 -X 800 -p 16 -x Bowtie_Index -1 R1.fastq -2 R2.fastq | samtools view -Sb.

### Transcript abundances

We used two different algorithms to compute abundances: an expectation-maximization algorithm (RSEM [15]) and an online method algorithm (eXpress [18]). As part of Trinity proposed pipeline (pipeline 1), RSEM was used to calculate isoform and gene abundances. The following parameters were used in the command line: run_RSEM_align_n_estimate.pl --transcripts Trinity.fasta --seqType fq --left R1.fq --right R2.fq. In pipeline 2, eXpress was coupled to bowtie2 aligner to calculate isoforms and genes abundances.

### Differential expression

The function of differential expression analysis is to point up isoforms or genes for which abundances changed significantly across experimental conditions. EdgeR [16], used in pipeline 1, handles the lack of biological replicate by simulating it, although the variance parameter was hard to evaluate. We chose to use 0.01 for this parameter since this is the value proposed for technical replicates. We ran EdgeR as part of the Trinity pipeline with the following command line: run_DE_analysis.pl --matrix counts.matrix --method edgeR –dispersion 0.01. EBSeq [19], used in pipeline 2, was developed specifically to counter biases in isoform differential expressions. We followed the EBSeq user manual instructions and used 15 iterations for convergence at a FDR of 5%.

## Competing interests

The authors declare that they have no competing interests.

## Authors’ contributions

EG carried out the bioinformatics calculations, wrote the manuscript, and designed figures and tables throughout the article. SJ designed the experiment and participated in writing the manuscript. Both authors read and approved the final manuscript.
